# The impact of microdosed plyometric training on speed and explosive abilities of football players during the pre-season

**DOI:** 10.1186/s13102-026-01556-5

**Published:** 2026-02-05

**Authors:** Marián Škorik, Tomáš Kalina, Martin Pupiš, Michal Hrubý

**Affiliations:** 1 Faculty of Sports Science and Health, Matej Bel University, Banská Bystrica, Slovakia; 2 Faculty of Sports Studies, Masaryk University, Brno, Czech Republic

**Keywords:** Microdosing, Plyometric training, Youth football, Reactive strength index, Countermovement jump, Sprint

## Abstract

**Background:**

Microdosed training distributes a given training stimulus into shorter, more frequent sessions. This study investigated whether a microdosed plyometric program produces similar adaptations to a traditional plyometric program when the total number of plyometric contacts is closely matched in elite youth football players.

**Methods:**

In this quasi-experimental, two-group study, twenty-four elite U19 players were allocated to a traditional training group (TRG, *n* = 12, 2 sessions·week⁻¹, ~ 40 min·session⁻¹) or a microdosed group (MDG, *n* = 12, 3–4 sessions·week⁻¹, ~ 20 min·session⁻¹). Allocation was nonrandomized and matched on countermovement jump (CMJ) height and modified reactive strength index (RSI mod) from a drop jump (DJ), with standing broad jump (SBJ) used as a tiebreaker. Total plyometric contact volume was closely matched over an 8-week intervention. Primary outcomes were 30 m sprint mechanical outputs (1080 Sprint: peak speed, peak force, peak power), DJ RSI mod, and CMJ metrics. Secondary outcomes were the 15–0–5 change of direction (CoD) test outputs (time, acceleration, and deceleration), and SBJ. Within-group pre–post changes were tested using paired t-tests and between-group comparisons using independent t-tests on change scores (α = 0.05). Nonparametric tests were conducted as sensitivity analyses. Effect sizes are reported as absolute Hedges’ g (|g|).

**Results:**

Primary outcomes improved in both groups: DJ RSI mod increased by 14.3% (TRG) and 12.5% (MDG), CMJ height by 4.5% and 9.6%, and 30 m sprint peak speed by 2.3% and 2.5% (within-group |g| = 0.02–1.09). Secondary outcomes were mixed across SBJ and 15–0–5 metrics (within-group |g| = 0.00–1.17). Between-group comparisons of change scores showed no evidence of differences (all *p* > 0.05).

**Conclusions:**

With closely matched total plyometric contacts, analyses showed no evidence that short-term adaptations differed between microdosed scheduling and a two-session format in U19 players. Microdosing may allow the same weekly dose to be delivered in shorter, more frequent sessions when scheduling is constrained.

**Trial registration:**

ClinicalTrials.gov (NCT07193706). Retrospectively registered on 18 September 2025.

**Supplementary Information:**

The online version contains supplementary material available at 10.1186/s13102-026-01556-5.

## Background

In professional sport, congested fixture periods shift priorities from performance gains to managing recovery efficiently and minimizing injury risk. Similar demands occur during major international tournaments, such as World Championships and EUROs, where fatigue has been reported when rest periods are shorter than three days [1, 2]. In this context, microdosing may help accommodate limited time and support recovery-management goals.

The concept of microdosing originated in pharmacology, where it refers to administering small doses of substances to minimize side effects and maximize efficacy [3, 4]. This concept has been applied to athletic training by distributing weekly volume across multiple sessions of varying size [5]. Afonso et al. [6] highlighted comparable methods, such as distributed training, which have been extensively utilized for motor learning [7]. Kilen et al. [8] examined how “micro-training” affects endurance and speed outcomes, concluding that short, frequent training sessions can be as effective as longer, less frequent sessions, provided that the total weekly training volume remains consistent. The microdosing approach has been implemented across training modalities, including resistance training [9], speed training [10], and plyometric training [11], with positive adaptations reported.

Plyometric training relies on the stretch-shortening cycle (SSC), which is essential for generating force during jumping, acceleration, and sprinting [12]. This is particularly important in football, as sprinting and explosive activities often precede goal situations [13]. The SSC consists of three phases: eccentric, isometric (amortization), and concentric [14, 15]. These phases involve neuromuscular and tendon-related adaptations [16–18], with evidence of altered muscle activation patterns during braking tasks [19]. Effective use of the SSC has been associated with improvements in muscle strength, explosiveness, sprinting ability, acceleration, and change of direction (CoD) [20–22], as well as coordination and tendon properties that support efficient high-speed movement [23, 24]. Additionally, plyometrics can be utilized for fatigue monitoring [25–27] and performance diagnostics. Standard indices include the eccentric utilization ratio (EUR) [28–31], the reactive strength index (RSI) [32, 33], and the modified reactive strength index (RSI mod) [34, 35], which quantify SSC-related performance and reactive abilities.

When combining microdosing with plyometric training, it is essential to consider the minimum effective dose (MED), which helps determine optimal training volume and identify the lowest training stimulus that still elicits the desired adaptations [36, 37]. Hansen [38] highlights that training doses are often influenced by organizational constraints, such as facility availability or league rules, rather than physiological needs. Torres-Banduc et al. [39] examined the minimum effective dose in plyometrics in a four-week study involving 44 participants. They reported that a lower training volume (approximately 100 contacts, reported as jumps in the original study, per session) significantly improved tendon viscoelastic properties, such as stiffness and Achilles’ tendon relaxation capacity. A greater volume (approximately 200 contacts) is required to increase muscle strength and power significantly. Overall, adaptations depend on volume, intensity, and frequency, with intensity classifications incorporating factors such as impact velocity and ground contact characteristics [40].

Cuthbert et al. [5] applied pharmacological concepts to microdosing in sport training, describing training volume as dose, intensity as potency, and the body’s response as a therapeutic effect. Afonso et al. [6] emphasized that achieving “true” microdosing requires a reduction in total training volume. Compared with traditional approaches, Cuthbert et al. [5] and Cuadrado-Peñafiel et al. [10] maintained the same training volume per cycle, whereas Liu, Wang, and Xu [11] reduced this volume. Bonder and Shim [41] demonstrated a practical application using short, intense training units (20–30 min) combining resistance training and plyometric exercises with a focus on speed, agility, and CoD. Given the ongoing debate regarding the term “microdosing,” we use it operationally to describe a programming strategy in which an equivalent weekly training dose is distributed across shorter, higher-frequency sessions rather than reduced in magnitude. This operationalization aligns with the distributed-practice principle (i.e., spacing practice exposures across time). However, in this manuscript, “distributed practice” is used only as a general concept for spacing, whereas “microdosing” refers to the applied load-management strategy of distributing an equivalent dose over a week. Accordingly, weekly plyometric contact volume was closely matched between conditions to isolate the effect of dose distribution from the impact of dose magnitude.

Based on previous findings [11], we hypothesized that microdosed plyometric training would lead to improvements in jumping performance and sprint-related abilities comparable to those of traditional plyometric training, despite the lower volume per session. The purpose of this study was to compare the effects of two distributions of an equivalent weekly plyometric dose (traditional vs. microdosed scheduling) on neuromuscular, speed, and power outcomes in elite youth football players. Evidence on microdosed plyometric scheduling in elite adolescent footballers remains limited, although congested training and competition demands are highly relevant in this population. We assessed maximum velocity, vertical jump height, reactive power, CoD speed, and deceleration ability, which are key performance outcomes in football.

## Methods

### Participants

The sample comprised 24 elite U19 football players (age, 17.26 ± 0.64 years; height, 181.60 ± 5.81 cm; body mass, 74.95 ± 7.04 kg) competing at the highest national level. All participants were recruited from a single elite youth football club through collaboration with the team coaches.

Players were allocated to the traditional training group (TRG) or the microdosed group (MDG) using a deterministic matching procedure. Matching was based on baseline countermovement jump (CMJ) height and modified reactive strength index (RSI mod) from the drop jump (DJ). When multiple matches were possible, standing broad jump (SBJ) performance was used as a tiebreaker because it reflects horizontal force production capacity. Players were ranked on the matching variables and alternately assigned to TRG or MDG. Baseline summaries were then reviewed to confirm similar values on the matching variables. The TRG (*n* = 12; age, 17.22 ± 0.70 years; height, 180.00 ± 6.54 cm; body mass, 73.41 ± 8.01 kg) trained twice per week. The MDG (*n* = 12; age, 17.33 ± 0.56 years; height, 184.28 ± 3.09 cm; body mass, 77.52 ± 4.29 kg) trained 3–4 sessions per week.

The purpose of deterministic allocation was to reduce baseline differences between groups. However, because allocation was based on jump performance and did not incorporate sprint or CoD outcomes, the groups may still differ systematically in sprint/CoD capacities and other unmeasured characteristics, resulting in residual confounding and limiting causal inference. Participants, coaches, and outcome assessors were not blinded to group allocation. The primary investigator conducted testing and group allocation but did not participate in the team’s training process during the intervention. Performance testing used standardized protocols and objective measurement systems to minimize tester-related bias.

Inclusion criteria were active club membership during the intervention and medical clearance for participation. Exclusion criteria were medical conditions precluding training or testing, training attendance < 75% of sessions, and failure to complete mandatory tests. Participants were experienced athletes with prior plyometric training and were familiar with the testing procedures. Additional familiarization was not required. No injuries occurred during the intervention.

### Experimental design

**Fig. 1:**
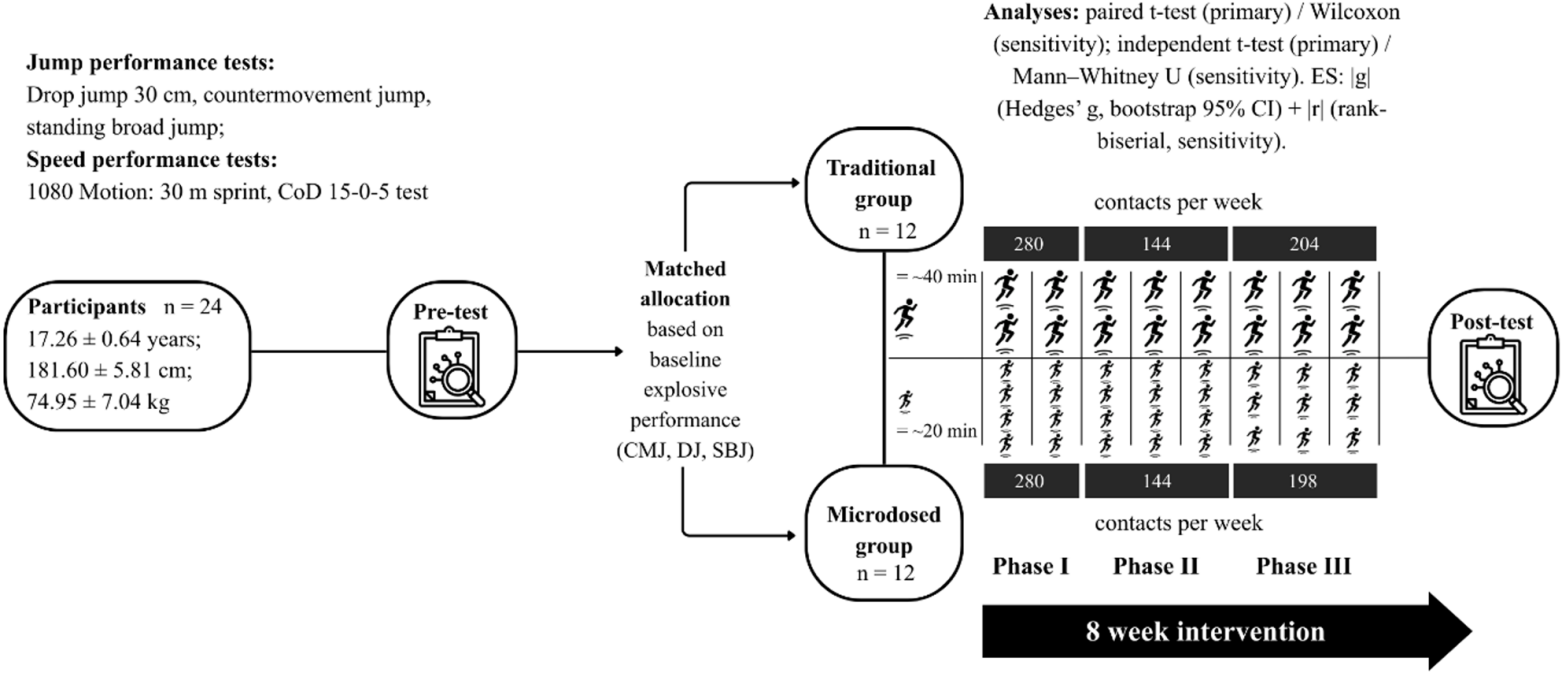
Study design and 8-week intervention comparing traditional vs. microdosed training. Note. TRG – traditional group; MDG – microdosed group; DJ – drop jump; CMJ – countermovement jump; SBJ – standing broad jump; CoD – change of direction. Players were assigned via matched allocation (based on baseline explosive performance) to a traditional group (TRG; n = 12) or a microdosed group (MDG; n = 12). Weekly training contact volume (contacts•week⁻¹) is shown across the three intervention phases

This study used a concurrent, two-group, quasi-experimental design conducted over an eight-week pre-season intervention. The TRG completed plyometric training twice a week, whereas the MDG completed it three to four times a week. The program was divided into three cycles, tailored to the needs of the U19 team, and developed in consultation with the strength and conditioning coach. All sessions were supervised and delivered by the team’s strength and conditioning coach in collaboration with the research team. Beyond the plyometric intervention, all players trained within a single U19 squad and completed the same club-prescribed technical and tactical sessions (four pitch-based sessions per week), strength and conditioning activities, and match schedule during the pre-season. The intervention aimed to isolate the effect of plyometric dose distribution (session frequency/spacing and per-session density) while keeping the broader training environment consistent across groups. External load and recovery were not objectively quantified (e.g., global positioning system metrics, session rating of perceived exertion, wellness assessment, or match minutes), so equivalence of individual load and recovery between groups cannot be confirmed. The overall study design and the 8-week intervention schedule are summarized in Fig. 1.

### Test procedures

Baseline testing was conducted at the club facilities. Speed tests (30 m sprint and 15–0–5 test) and the SBJ were performed outdoors on an artificial turf area where the players regularly trained. Jump tests were performed indoors in the club gym. Post-intervention testing was conducted at the exact locations and under similar conditions as the baseline testing, with the only difference being the timing of the test. Before testing, participants completed a standardized 15-minute warm-up based on the Raise-Activation-Mobilization-Potentiation (RAMP) protocol to prepare for the testing load [42].

### Speed tests

Speed testing included a 30 m sprint and a 15–0–5 deceleration and CoD test. Both tests were performed using a 1080 Sprint (1080 Motion, Västerås, Sweden) in isotonic mode. For the 30 m sprint, a 1 kg resistance setting was used to enable measurement of speed and acceleration across the sprint. Participants began in a high two-point stance and initiated the sprint at a self-selected moment. The 1080 Sprint device was selected due to its reliability and accuracy [43]. From the trials, we extracted peak speed (PS), peak force (PF), and peak power (PP). Each player completed two sprint trials, and the trial with the greater peak velocity was retained for analysis.

The 15–0–5 CoD and deceleration test was divided into two phases. The first 15 m was Phase A, during which maximum acceleration (Max acc A) and maximum deceleration (Max dec A) were assessed. The subsequent 5 m after the turn was Phase B, during which maximum reacceleration (Max acc B) was evaluated [44]. The test was performed in isotonic mode using 3 kg for acceleration and deceleration, and 3 kg for reacceleration. Each player performed two trials, one with a 180° turn to the right and one with a 180° turn to the left. The best trial was used for the final analysis. The test has demonstrated high reliability for assessing kinetic and kinematic data [45], and its relevance is supported by its ability to capture deceleration demands in game situations [44].

### Diagnostics of vertical force production

Vertical force production was assessed using the CMJ on K-Delta strain gauge force plates (Kinvent, Orsay, France) in a bilateral setup [46]. Participants performed two trials, and the best trial was used for analysis. Outcomes included jump height (JH), relative force (RF), and relative power (RP). Relative parameters were normalized to body mass. Reactive performance was assessed using a 30 cm drop jump on a dual wireless force-plate system (Hawkin Dynamics, Westbrook, USA) [47]. Reactive strength outcomes were reported as modified reactive strength index (RSI mod), calculated as jump height divided by ground contact time (as defined by the Hawkin Dynamics system). Two force-plate systems were used due to equipment availability. Protocols were standardized across baseline and follow-up testing.

### Horizontal explosive force

The SBJ was assessed with a measuring tape. Participants performed two bilateral trials and one unilateral trial on each leg. Distances were recorded to the nearest 1 cm. A no-countermovement instruction was used to minimize technical variability. Montalvo et al. [48] suggested that bilateral broad jumps are effective in predicting acceleration performance over 5–30 m, supporting the diagnostic value of the test.

### Intervention training protocol

The intervention lasted eight weeks, consistent with recommendations for adaptation duration [49] and the typical length of the pre-season period in youth football. The MDG completed a microdosed plyometric program (3–4 sessions per week, ~ 20 min per session), whereas the TRG completed a traditional program (2 sessions per week, ~ 40 min per session). The macrocycle was divided into three phases. Phase 1 (2 weeks) included 208 contacts per week and served as a volume-accumulation phase with lower-intensity contacts. Phase 2 (3 weeks) reduced weekly volume to 144 contacts and included more demanding reactive exercises (e.g., pogo hops and drop jumps). In Phase 3 (3 weeks), the MDG frequency decreased from four to three sessions per week due to the start of the school year, which reduced the planned session-frequency contrast despite matched weekly contacts. The microdosed structure was preserved by spreading the contacts across three shorter sessions rather than two longer sessions. Exercise selection and phase allocation are shown in Table 1. Because the number of contacts varied across sessions within the same week, per-session contact volume is reported in Table 2 as a range rather than a mean. Attendance was monitored at each session.

**Table 1:** Exercise selection and weekly distribution of plyometric drills across phases

Exercises	Traditional training group			Microdosed training group		
	Phase 1	Phase 2	Phase 3	Phase 1	Phase 2	Phase 3
*Pogo hops (Unil.)*	1 × 4 × 10	1 × 2 × 6 (EA)		1 × 4 × 10	1 × 2 × 6 (EA)	
*CMJ box jump (Unil.)*	1 × 4 × 6		1 × 2 × 6 (EA)	1 × 4 × 6		1 × 2 × 6 (EA)
*Unil. broad jump*	1 × 4 × 6 (EA)			2 × 2 × 6 (EA)		
*Lateral box hop*	1 × 2 × 6 (EA)			1 × 2 × 6 (EA)		
*Medial box hop*	1 × 2 × 6 (EA)			1 × 2 × 6 (EA)		
*Lateral bound*	1 × 4 × 6 (EA)			2 × 2 × 6 (EA)		
*Hurdle hops*		1 × 2 × 6 (EA)	1 × 2 × 6 (EA)		1 × 2 × 6 (EA)	
*Unil. split jumps*		1 × 2 × 6 (EA)			2 × 1 × 6 (EA)	
*Drop Jump 30 cm (Unil. -15 cm)*		1 × 4 × 6	1 × 2 × 6 (EA)		2 × 2 × 6	1 × 3 × 6 (EA)
*Hurdle hops medial paused*		1 × 2 × 6 (EA)			1 × 2 × 6 (EA)	
*Hurdle hops medial continuous*			1 × 2 × 6 (EA)			1 × 2 × 6 (EA)
*Hurdle hops lateral paused*		1 × 2 × 6 (EA)			1 × 2 × 6 (EA)	
*Hurdle hops lateral continuous*			1 × 2 × 6 (EA)			
*Linear bounds*			1 × 2 × 6 (EA)			1 × 3 × 6 (EA)
*Triple broad jump*			1 × 2 × 3 (EA)			1 × 3 × 3 (EA)
*Unil. tuck jump*			1 × 2 × 6 (EA)			1 × 2 × 6 (EA)

Note. *CMJ* Countermovement jump, *Unil.* Unilateral, *EA* (each) indicates that the indicated number of repetitions was performed on each limb for unilateral exercises. Training volume notation is provided in the format: 1 × 2 × 6, where the first number indicates how many times per week the exercise was performed, the second number indicates the number of sets, and the third number indicates repetitions per set

**Table 2:** Weekly plyometric contact distribution by phase and group

Phase	Ses/w TRG	Ses/w MDG	CPS TRG	CPS MDG	TCW TRG	TCW MDG
1	2	4	96–112	48–64	208	208
2	2	4	72	36	144	144
3	2	3	96–108	60–78	204	198

Note. *Ses/w* Sessions per week, *CPS* Contacts per session, *TCW* Total contacts per week. Contacts per session are reported as ranges because session contact counts varied within each week. One “contact” was defined as one ground contact per repetition; unilateral drills were counted per limb (EA), and one triple broad jump repetition was counted as three ground contacts; TCW values reflect the fixed weekly target; CPS ranges indicate the minimum–maximum per-session contacts within that week, distributed across sessions to sum to the TCW

### Statistical analyses

The data were processed and analyzed using JASP Statistics (version 0.19.2.0, The Netherlands) and custom Python (Python Software Foundation) scripts. Prior to analysis, all exported outputs were screened for plausibility and obvious recording artefacts. Invalid trials were replaced with the alternative trial from the same testing session, and all variables were cross-checked against the raw exports to minimize transcription errors. Descriptive statistics (mean ± standard deviation) summarized somatic characteristics and outcomes at baseline and post-intervention. No a priori power analysis was conducted, and the sample size reflected the number of eligible U19 players available during the study period.

Within-group pre–post changes were analyzed using paired-samples t-tests. Between-group differences were analyzed using independent-samples t-tests applied to change scores (post minus pre). Given the small sample size and occasional extreme responders, non-parametric tests were performed as sensitivity analyses, using the Wilcoxon signed-rank test for within-group comparisons and the Mann–Whitney U test for between-group comparisons of change scores. All tests were two-tailed with α = 0.05.

For consistency across outcomes, Hedges’ g values are reported as absolute magnitudes (|g|), with the direction inferred from the mean change (or group difference in change). For within-group comparisons, Hedges’ g was computed from paired pre–post difference scores. For between-group comparisons, the calculation was based on the differences in change scores between groups. The small-sample correction J = 1 − 3/(4df − 1) was applied, with df defined by the corresponding comparison. Percentile bootstrap 95% CIs for |g| were estimated with 6000 resamples (seed = 123), resampling paired observations within participants for within-group effects and resampling within groups for between-group effects. Absolute rank-biserial correlations (|r|) from Wilcoxon/Mann–Whitney sensitivity analyses are reported in Supplementary Table S1 and were not used for primary inference.

Percentage changes were calculated by expressing the difference between post- and pre-intervention group means relative to the pre-intervention mean, multiplied by 100. Effect sizes were interpreted using conventional thresholds for Hedges’ g (|g|: small 0.20–0.49, medium 0.50–0.79, large ≥ 0.80), with values below 0.20 considered trivial [50]. Given the small sample size, these labels were treated as secondary to the magnitude and uncertainty of the estimates.

## Results

All 24 players were included in the final dataset and in all within-group and between-group analyses. Matching was based on jump performance (CMJ height and DJ RSI mod) rather than sprint or CoD outcomes. Baseline group comparisons using Welch’s t tests showed no evidence of differences across outcomes (all *p* ≥ 0.157; Table 3). In Phase 3, the MDG frequency was reduced from four to three sessions per week due to the start of the school year. Total weekly contact volume remained closely matched across groups (Phase 3: TRG 204 vs. MDG 198 contacts·week⁻¹; Table 2). Within-group improvements were observed across multiple performance parameters in both groups. Detailed descriptive statistics for these outcomes, including pre- and post-intervention means, standard deviations, and within-group effect sizes, are presented in Table 4. Between-group tests on change scores provided no evidence of differences across outcomes (*p* = 0.052–0.879). Non-parametric sensitivity analyses yielded comparable inferences (Supplementary Table S1).

**Table 3:** Baseline group comparisons for performance outcomes

Performance parameter	TRG (*n* = 12)	MDG (*n* = 12)	*p*-value¹
RSI mod	2.38 ± 0.35	2.45 ± 0.48	0.697
CMJ jump height	33.42 ± 2.35	33.75 ± 3.82	0.800
CMJ relative force	2.77 ± 0.39	2.79 ± 0.25	0.912
CMJ relative power	51.87 ± 3.35	51.50 ± 5.76	0.848
Broad jump bilateral	243.58 ± 9.45	244.92 ± 13.47	0.782
Broad jump left	215.17 ± 14.06	218.00 ± 16.28	0.653
Broad jump right	214.42 ± 13.10	215.58 ± 13.35	0.831
Peak Speed [m·s^−1^]	8.68 ± 0.31	8.82 ± 0.24	0.221
Peak Force [N]	41.84 ± 4.72	41.86 ± 2.70	0.989
Peak Power [W]	359.49 ± 44.83	365.54 ± 29.53	0.701
Time 15-0-5 [s]	4.83 ± 0.20	4.69 ± 0.26	0.157
Max Acc Phase A [m·s^−2^]	6.17 ± 0.57	6.22 ± 1.38	0.911
Max Dec Phase A [m·s^−2^]	7.69 ± 0.64	7.92 ± 0.64	0.377
Max Acc Phase B [m·s^−2^]	5.92 ± 0.47	5.90 ± 0.75	0.962

Note. Values are presented as mean ± SD

¹Between-group comparisons at baseline were conducted using Welch’s t-test. Given the small sample size, non-significant p-values should be interpreted as no evidence of difference rather than proof of equivalence

**Table 4:** Descriptive statistics of performance tests in traditional and microdosed groups before and after intervention

Performance Test	Performance parameter	Traditional (*n* = 12)				Microdosed (*n* = 12)			
		(Mean ± SD)		Hedges’ |g|	95% CI	(Mean ± SD)		Hedges’ |g|	95% CI
		Post	Post		(low, high)	Pre	Post		(low, high)
*Drop jump 30 cm*	*RSI mod*	2.38 ± 0.35	*2.72 ± 0.46	0.92	(0.59, 1.54)	2.45 ± 0.48	*2.76 ± 0.33	0.59	(0.09, 1.76)
*Counter-movement jump test*	*Jump height [cm]*	33.42 ± 2.35	*34.92 ± 2.31	0.72	(0.33, 1.45)	33.75 ± 3.82	*37.00 ± 4.41	0.71	(0.50, 1.39)
	*Relative force [N·kg* ^*−1*^ *]*	2.77 ± 0.39	2.80 ± 0.17	0.07	(0.01, 0.87)	2.79 ± 0.25	2.79 ± 0.27	0.02	(0.01, 0.70)
	*Relative power [W·kg* ^*−1*^ *]*	51.87 ± 3.35	53.51 ± 3.56	0.54	(0.07, 1.37)	51.50 ± 5.76	*55.53 ± 4.31	0.69	(0.29, 2.00)
*Standing broad jump*	*Broad jump bilateral [cm]*	243.58 ± 9.45	248.00 ± 10.73	0.57	(0.08, 1.92)	244.92 ± 13.47	246.08 ± 12.24	0.09	(0.01, 0.67)
	*Broad jump left [cm]*	215.17 ± 14.06	*222.42 ± 10.40	0.63	(0.13, 1.40)	218.00 ± 16.28	216.67 ± 11.66	0.13	(0.01, 0.83)
	*Broad jump right [cm]*	214.42 ± 13.10	219.25 ± 10.59	0.36	(0.02, 1.41)	215.58 ± 13.35	*223.08 ± 9.96	0.70	(0.25, 1.47)
*30 m sprint*	*Peak speed [m·s* ^*−1*^ *]*	8.68 ± 0.31	*8.87 ± 0.25	1.09	(0.68, 2.04)	8.82 ± 0.24	*9.04 ± 0.25	0.92	(0.55, 1.65)
	*Peak force [N]*	41.84 ± 4.72	*44.73 ± 3.15	0.63	(0.11, 1.88)	41.86 ± 2.70	*45.42 ± 2.44	0.89	(0.45, 1.74)
	*Peak power [W]*	359.49 ± 44.83	*391.25 ± 35.80	0.78	(0.24, 2.11)	365.54 ± 29.53	*403.87 ± 23.08	0.97	(0.60, 1.69)
	*Time 15-0-5 [s]*	4.83 ± 0.20	4.76 ± 0.25	0.44	(0.04, 1.23)	4.69 ± 0.26	4.69 ± 0.27	0.00	(0.00, 0.73)
*15-0-5 test*	*Max acc phase A [m·s* ^*−2*^ *]*	6.17 ± 0.57	6.38 ± 1.24	0.19	(0.01, 0.85)	6.22 ± 1.38	6.18 ± 0.95	0.02	(0.01, 0.87)
	*Max dec phase A [m·s* ^*−2*^ *]*	7.69 ± 0.64	7.90 ± 0.60	0.33	(0.02, 1.12)	7.92 ± 0.64	*8.46 ± 0.52	1.17	(0.63, 2.49)
	*Max acc phase B [m·s* ^*−2*^ *]*	5.92 ± 0.47	*6.41 ± 0.41	0.83	(0.32, 1.99)	5.90 ± 0.75	*6.30 ± 0.47	0.73	(0.25, 1.54)

Note. *RSI mod* Modified reactive strength index, *Acc* Acceleration, *Dec* Deceleration, *CI* Confidence interval, *W* Watts, *N* Newtons, *kg* Kilograms, *m* Meters, *s* Seconds, *ES* Effect size

Hedges’ g is reported as |g|; 95% CIs were estimated via bootstrap (6000 resamples)

* – represents p < 0.05 for pre vs. post within-group comparisons (paired t-test)

### Countermovement jump

Jump height increased in both groups (TRG: *p* < 0.05, g = 0.72; MDG: *p* < 0.05, g = 0.71). CMJ relative force showed no statistically significant change in either group (TRG: *p* > 0.05, g = 0.07; MDG: *p* > 0.05, g = 0.02). CMJ relative power increased in the MDG (*p* < 0.05, g = 0.69), while the TRG showed no statistically significant change (*p* > 0.05, g = 0.54). Between-group comparisons of change scores showed no evidence of a difference for CMJ jump height (*p* > 0.05, g = 0.51), CMJ relative force (*p* > 0.05, g = 0.06), or CMJ relative power (*p* > 0.05, g = 0.53).

### Drop jump (30 cm)

RSI mod improved significantly in both groups (TRG: *p* < 0.05, g = 0.92; MDG: *p* < 0.05, g = 0.59). The between-group comparison of change scores showed no evidence of a difference (*p* > 0.05, g = 0.08). 

### Standing broad jump

**Fig. 2:**
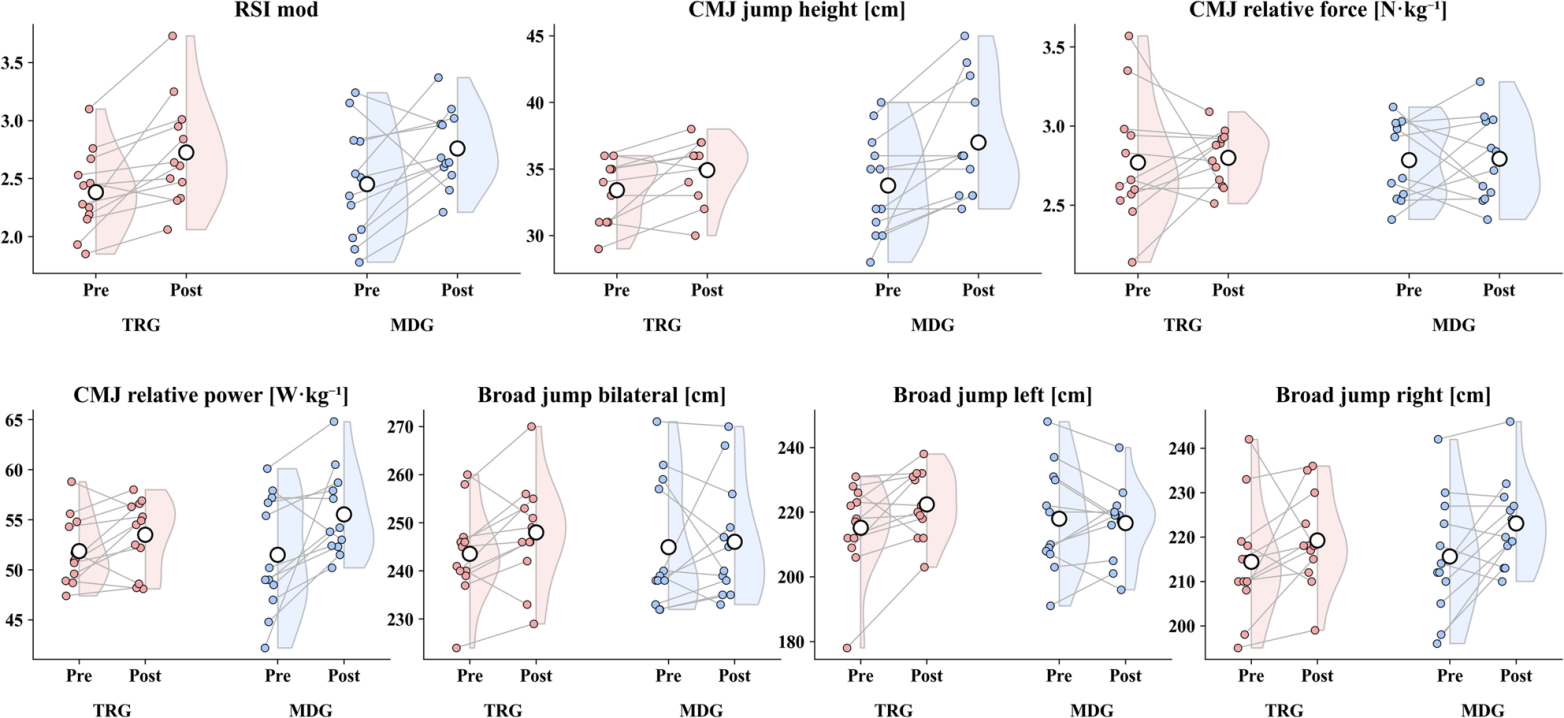
Changes in jump performance and CMJ-derived variables from pre- to post-intervention in TRG and MDG. Note. RSI mod – modified reactive strength index; CMJ – countermovement jump. Colored points represent individual participants, and grey lines connect paired pre- and post-values. Shaded half-violin plots were used to visualize the distribution of values within each group; open circles indicate group means. TRG (red) and MDG (blue) denote the two study groups (as defined in the Methods)

For SBJ bilateral, neither group showed a statistically significant pre-post change (TRG: *p* > 0.05, g = 0.57; MDG: *p* > 0.05, g = 0.09). For SBJ left, TRG improved (*p* < 0.05, g = 0.63), whereas MDG showed no statistically significant change (*p* > 0.05, g = 0.13). For SBJ right, MDG improved (*p* < 0.05, g = 0.70), while TRG showed no statistically significant change (*p* > 0.05, g = 0.36). Between-group comparisons of change scores showed no evidence of a difference for SBJ bilateral (*p* > 0.05, g = 0.32) and SBJ right (*p* > 0.05, g = 0.23). For SBJ left, the between-group difference did not reach statistical significance (*p* > 0.05, g = 0.81). Jump-related pre–post changes are visualized in Fig. 2.

### 30 m linear sprint (1080 Sprint)

**Fig. 3:**
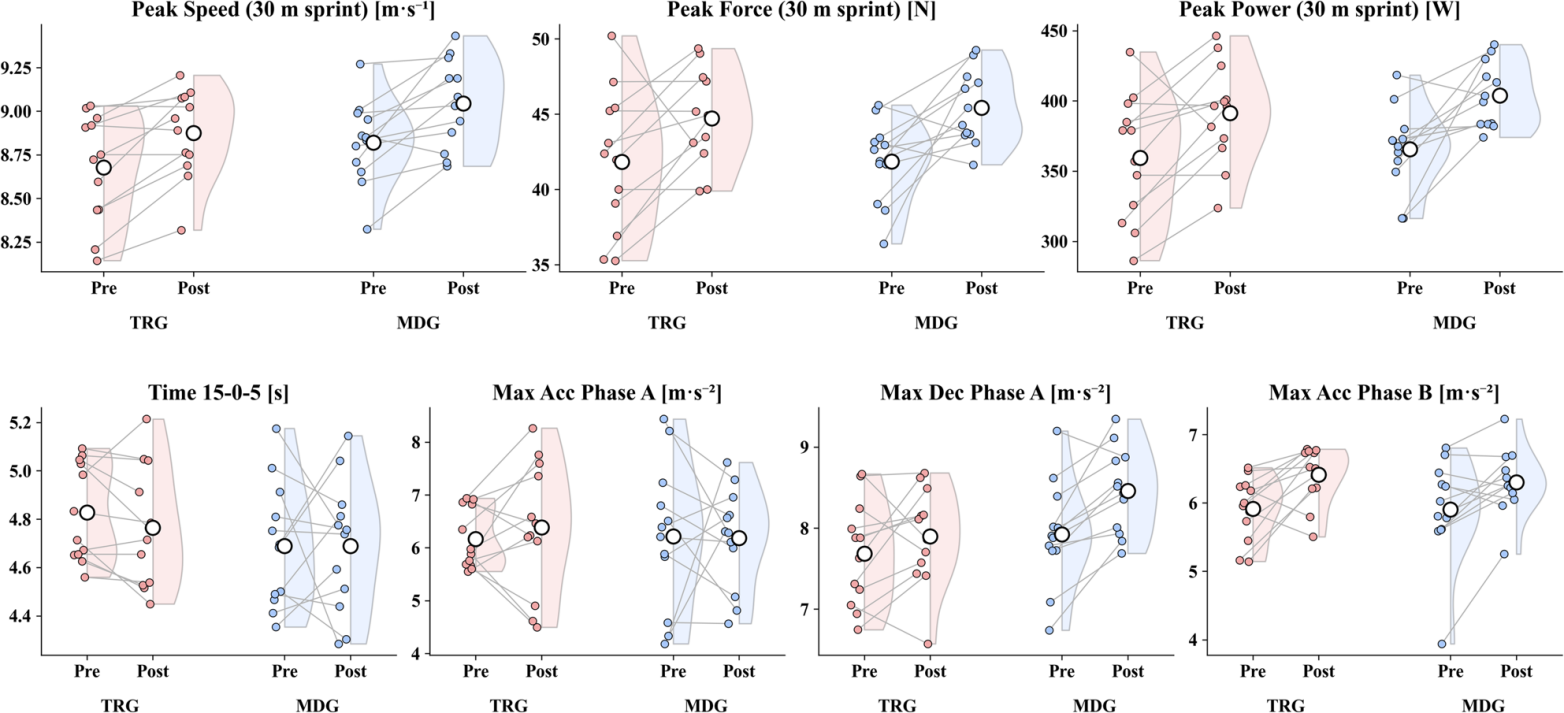
Changes in sprint performance and sprint-derived mechanical variables from pre- to post-intervention in TRG and MDG. Note. Acc – acceleration; Dec – deceleration. Colored points represent individual participants, and grey lines connect paired pre–post values. Shaded half-violin plots were used to visualize the distribution of values within each group; open circles indicate group means. TRG (red) and MDG (blue) denote the two study groups (as defined in the Methods). For 15–0–5 time, lower values indicate better performance

Peak speed (PS), peak force (PF), and peak power (PP) increased in both groups (all *p* < 0.05), with effect sizes as follows: TRG: PS g = 1.09, PF g = 0.63, PP g = 0.78; MDG: PS g = 0.92, PF g = 0.89, PP g = 0.97. Between-group comparisons of change scores showed no evidence of a difference for PS (*p* > 0.05, g = 0.13), PF (*p* > 0.05, g = 0.16), or PP (*p* > 0.05, g = 0.17). These sprint-related pre–post changes are visualized in Fig. 3.

### Change of direction and mechanics: 15–0–5 test

In TRG, total time decreased but was not statistically significant (*p* > 0.05, g = 0.44). Phase A maximum acceleration and maximum deceleration showed no statistically significant change (Max Acc: *p* > 0.05, g = 0.19; Max Dec: *p* > 0.05, g = 0.33), whereas Phase B maximum acceleration increased (*p* < 0.05, g = 0.83). In MDG, total time showed no change (g = 0.00), Phase A maximum acceleration showed no change (*p* > 0.05, g = 0.02), Phase A maximum deceleration increased (*p* < 0.05, g = 1.17), and Phase B maximum acceleration increased (*p* < 0.05, g = 0.73). Between-group comparisons of change scores showed no evidence of a difference for any parameter (all *p* > 0.05). A medium between-group effect estimate was observed for Δ Phase A maximum deceleration (*p* > 0.05, g = 0.60), and the CI spanned small-to-large effects (Table 5). No additional subgroup or exploratory analyses were conducted beyond the predefined outcomes.

**Table 5:** Comparison of pre-post percentage changes and between-group differences in performance parameters

Performance Test	Performance parameter	Δ Pre – Post (%)		*p*	Hedges’ |g|	95% CI (low, high)	Interpretation
		Traditional (*n* = 12)	Microdosed (*n* = 12)				
Drop jump 30 cm	*RSI mod*	14.30	12.50	0.843	0.08	(0.01, 0.97)	trivial
Countermovement jump test	*Jump height [cm]*	4.49	9.63	0.207	0.51	(0.04, 1.16)	medium
	*Relative force [N·kg* ^*−1*^ *]*	1.05	0.24	0.879	0.06	(0.01, 1.04)	trivial
	*Relative power [W·kg* ^*−1*^ *]*	3.15	7.83	0.189	0.53	(0.04, 1.28)	medium
Standing broad jump	*Broad jump bilateral [cm]*	1.81	0.48	0.422	0.32	(0.02, 1.37)	small
	*Broad jump left [cm]*	3.37	-0.61	0.052	0.81	(0.12, 1.81)	large
	*Broad jump right [cm]*	2.25	3.48	0.569	0.23	(0.02, 1.05)	small
30 m Sprint	*Peak Speed [m·s* ^*−1*^ *]*	2.27	2.54	0.744	0.13	(0.01, 0.97)	trivial
	*Peak Force [N]*	6.90	8.50	0.685	0.16	(0.01, 0.97)	trivial
	*Peak Power [W]*	8.83	10.49	0.669	0.17	(0.01, 0.98)	trivial
15-0-5 test	*Time 15-0-5 [s]*	-1.31	0.00	0.539	0.25	(0.01, 1.21)	small
	*Max Acc Phase A [m·s* ^*−2*^ *]*	3.49	-0.55	0.649	0.18	(0.01, 1.16)	trivial
	*Max Dec Phase A [m·s* ^*−2*^ *]*	2.76	6.76	0.143	0.60	(0.05, 1.50)	medium
	*Max Acc Phase B [m·s* ^*−2*^ *]*	8.39	6.73	0.654	0.18	(0.01, 1.07)	trivial

Note. *Δ Pre–Post (%)* Descriptive percentage change from pre to post. *RSI mod* Modified reactive strength index, *Acc* Acceleration, *Dec* Deceleration, *CI* Confidence interval, *W* Watts, *N *Newtons, *kg* Kilograms, *m* Meters, *s *Seconds, *ES* Effect size

Between-group p-values are from t-tests on change scores (Post–Pre); Hedges’ g is reported as |g| with bootstrap 95% CIs (6000 resamples). For 15–0–5 time, negative Δ Pre–Post (%) indicates faster performance

## Discussion

Across the intervention, both groups improved in several performance outcomes, which may reflect concurrent team training and typical pre-season adaptation. With weekly plyometric contact volume matched between conditions, between-group analyses provided no evidence of a difference in the magnitude of change. This should not be interpreted as evidence of equivalence between formats. Accordingly, distributing the same weekly plyometric volume across more frequent, shorter sessions may be a feasible strategy in settings where logistical constraints limit session duration. Here, “microdosing” refers to a higher-frequency distribution of an equivalent weekly training dose, rather than a reduced weekly dose. This scheduling flexibility may be particularly relevant during congested microcycles, when recovery time is limited, and fatigue management is critical [2]. Injury risk has also been reported to be higher during periods of fixture congestion [51]. In such contexts, maintaining speed-power qualities may benefit from more frequent, lower-density exposures, consistent with the relatively short residual training effect typically reported for maximal speed [52]. Given that simple wellness markers (sleep, stress, fatigue, soreness) show meaningful relationships with training load in football, integrating brief daily monitoring may be especially important when distributing work across more frequent sessions [53].

Although our intervention was conducted in pre-season, evidence from microdosed sprint-load distribution suggests that manipulating within-week distribution can support sprint-related mechanical outputs while aiming to minimize fatigue [10]. Thus, microdosing may be a practical option when weekly scheduling or matching congestion limits the feasibility of higher-density sessions. Because concurrent football training was not quantified, individual differences in running demands, match minutes, and recovery could have varied across players and may have diluted or masked minor effects attributable solely to plyometric dose distribution. Consequently, the null between-group findings reflect squad-standardized training exposure. Individual differences in external load could have masked small distribution effects. The findings are limited to elite U19 male players from a single club and may not generalize to female athletes, other age groups, different competitive levels, or in-season phases.

Plyometric training is associated with neuromuscular adaptations that can enhance SSC performance, including improved neural drive and voluntary activation, as well as more efficient intra- and intermuscular coordination [12, 17, 24, 54]. Behrens et al. [54] reported changes in the neural and mechanical properties of the knee extensors following an 8-week plyometric intervention, supporting a neuromuscular contribution to performance gains. Plyometric tasks can increase muscle activation and refine movement-specific neuromuscular patterning, although the specific mechanisms likely depend on the exercise selection, intensity, and the athlete’s training status [55]. Computational work has also proposed plausible neuromuscular pathways through which plyometric and strength stimuli could shape strength-speed outcomes, but these inferences should be interpreted as model-based hypotheses rather than direct experimental evidence [56].

In youth football, Bedoya et al. [23] recommend implementing plyometric training twice per week for 8–10 weeks, beginning with ~ 50–60 foot contacts and progressing to no more than ~ 80–120 contacts per session, with ~ 72 h between plyometric days, typically using 3–4 exercises performed for 2–4 sets of 6–15 repetitions. In the present study, weekly exposure ranged from 144 to 208 contacts, with per-session densities of 72–112 (TRG) and 36–78 (MDG), placing TRG near the upper recommended per-session range. At the same time, MDG remained below it, despite matched weekly exposure. Notably, this comparison spans different contexts. Bedoya et al. [23] synthesize youth football prescriptions typically applied to younger cohorts and a fixed two-session structure, whereas our intervention was delivered to elite U19 players during the pre-season, contrasting frequency and spacing while equating weekly contact dose. Additionally, training intensity and stimulus characteristics were periodized across the 8 weeks, allowing for distribution effects to be tested under a progressing stimulus rather than a static program. This may partly explain why frequency alone did not yield a clearly detectable added benefit once the weekly dose was sufficient. Meta-analytic evidence in young male football players suggests that plyometric jump training enhances jump and sprint outcomes, and that interventions lasting more than 7 weeks and comprising more than 14 sessions yield greater improvements in 10 m sprint performance [12]. This aligns with our 8-week intervention. Although the meta-analysis emphasizes 10 m sprint performance, we similarly observed improvements in 30 m sprint performance in our cohort. Chen et al. [24] also reported statistically significant improvements in adolescent athletes for both jumping outcomes and short sprint performance. Taken together, these comparisons suggest that our weekly contact volumes and duration exceeding 7 weeks were likely sufficient to elicit adaptation, which may reduce the likelihood of a clearly detectable additional benefit from within-week distribution when the total dose is equated [12]. This weekly range also aligns with volumes associated with more favorable outcomes related to stiffness. It remains below ranges that may be counterproductive when weekly jump volume becomes excessive (< 250 vs. >500 jumps·week⁻¹) [57].

Quantitative thresholds for a plyometric minimum effective dose remain unclear [58]. We matched weekly plyometric contact volume to isolate distribution effects, yet emerging studies suggest that favorable strength–speed adaptations may occur even at lower weekly doses, highlighting uncertainty around dose thresholds in plyometric programming [11, 59]. For example, Liu et al. reported similar RSI improvements under the regPJT and microPJT formats (11.7% vs. 12.0%) [11], which is consistent with our RSI-derived changes (RSI mod: 14.30% vs. 12.50%). A similar pattern was observed for CMJ (Liu: 6.4% vs. 4.3% under regPJT vs. microPJT [11]; present study: 4.49% vs. 9.63% in TRG vs. MDG, respectively).

Equating contacts does not guarantee equivalence in mechanical intensity or stimulus quality, because intensity and stimulus quality can differ despite identical contact counts. Plyometric intensity was periodized using key determinants (impact/force characteristics, ground contact time, limb support strategy) [40], progressing from predominantly bilateral and box-assisted variants toward unilateral drills with shorter ground contact times. Two complementary mechanisms may explain the lack of a between-group difference. First, both formats may have exceeded a practical “minimum sufficient” stimulus once weekly exposure was equated, consistent with dose-response patterns [57]. Second, a quality-fatigue trade-off may partly explain similar outcomes. Dense sessions can show fatigue-related kinetic alterations and small CMJ decrements as volume accumulates [58] and are linked to peripheral fatigue affecting force and RFD [60]. In contrast, a higher frequency may increase residual fatigue or muscle soreness throughout the week [61] and delayed SSC performance decrements [62], resulting in similar net adaptations.

In speed-related outputs, both groups showed within-group improvements consistent with the literature [12, 22–24]. Because sprint and CoD outcomes were not part of the matching procedure, these findings remain vulnerable to residual confounding. They should not be interpreted as demonstrating equivalence between formats for sprint or CoD performance.

Mechanistically, plyometric training may support sprint-related qualities through neural adaptations and more efficient SSC utilization [23, 24], and frequency-based distribution approaches have been linked to speed improvements in other contexts [10]. However, these findings must be interpreted alongside the concurrent pre-season football program: both groups completed the same team training, and external load was not monitored, meaning that speed-related gains may partly reflect non-plyometric stimuli (e.g., small-sided games), which have been shown to improve speed and related performance qualities in elite football settings [63].

In the 15–0–5 test, changes in completion times were small, which may reflect the absence of training specifically targeting braking and reacceleration mechanics. Similar modest responses in CoD outcomes have been reported in younger team-sport athletes [64]. RSI-derived metrics have been linked to deceleration-related performance qualities in team sports [32], suggesting some shared underpinning neuromuscular capacities. Nevertheless, any observed changes in deceleration and reacceleration should be interpreted cautiously, because without a football-only control condition, the effects of plyometric training cannot be separated from typical pre-season adaptation and concurrent training exposure.

### Study limitations

The primary limitation is the absence of a no-plyometric (football-only) control group. Without such a condition, within-group improvements cannot be attributed exclusively to the plyometric intervention and may partly reflect normal pre-season adaptation and concurrent team training. Second, group allocation was non-random and matching was restricted to baseline jump performance. Because allocation did not account for sprint and CoD outcomes, or other potential confounders (e.g., playing role, training history, neuromuscular profile), residual confounding, particularly for sprint and CoD outcomes, cannot be ruled out. Blinding of players and coaching staff was not feasible, and expectancy or motivational effects cannot be ruled out. However, outcomes were assessed using standardized protocols and objective measurement systems, reducing assessor-related measurement bias.

Third, external load and recovery were not monitored, which is particularly relevant when comparing different weekly distributions of the same plyometric dose. Psychological and well-being factors were also not assessed and may have influenced training tolerance and responsiveness across scheduling formats. Additionally, weekly plyometric “contacts” were used as a proxy for dose. However, contact counts do not ensure equivalence of mechanical intensity or stimulus quality (e.g., unilateral vs. bilateral demands, ground contact time, landing strategy, or impact characteristics), which limits mechanistic inference.

The relatively small sample size limits the statistical power to detect small to moderate between-group effects. This increases the risk of Type II error, particularly for sprint and CoD outcomes. Accordingly, null between-group findings should be interpreted as no evidence of a difference rather than evidence of equivalence. The intervention schedule also did not maintain a constant frequency throughout all phases (the microdosed group reduced the frequency from four to three weekly sessions in the final phase), which may have diluted frequency-specific effects and complicated the interpretation of a “high-frequency” exposure. Generalizability is limited to elite U19 male football players from a single club during a pre-season period, and extrapolation to female athletes, other age groups, different competitive levels, or in-season phases should be made cautiously. Although U19 players are typically post-PHV, maturity status was not assessed. Therefore, residual late-adolescent growth and maturity-related variability cannot be entirely excluded as contributors to the observed changes [65]. Finally, testing was conducted only immediately after the intervention. Delayed adaptations or retention effects were not assessed, and follow-up testing would strengthen conclusions regarding sustainability.

### Practical application

A microdosed format can be useful during congested microcycles to reduce per-session plyometric density and support execution quality, whereas a traditional two-session format may be more practical during less congested pre-season weeks when recovery days can be protected. Coaches should therefore select the format based on scheduling constraints and athlete readiness rather than expecting frequency alone to produce systematically superior outcomes when weekly dose is equated. Regardless of format, practitioners should individualize progressions (exercise selection, intensity, and contact distribution) and monitor training load and recovery where possible to manage fatigue and maintain movement quality.

## Conclusion

In elite U19 male football players during the pre-season, distributing weekly plyometric contact volume across more frequent sessions did not show clear advantages over a traditional two-session schedule for the measured outcomes. Both groups showed improvement in several outcomes over the intervention period, although no statistically significant between-group differences were detected. Accordingly, the results should be interpreted as indicating no clear evidence of a difference, rather than proof of equivalence. Practically, microdosing may be most useful when scheduling constraints or congested fixture periods limit the feasibility of more extended sessions, allowing coaches to maintain exposure while reducing per-session load and potentially managing fatigue. These results highlight the feasibility of a higher-frequency, lower session volume strategy, although stronger causal inferences require controlled designs and larger samples. Given the limited statistical power, training choices should consider individual needs, overall training load, and psychological load as part of a holistic approach to athlete management. Future research should include a no-plyometric control condition, objective monitoring of concurrent training load, athlete-reported psychological load/well-being, and longer follow-up to evaluate the sustainability of adaptations.

## Supplementary Information

Supplementary Material 1

## Data Availability

The data supporting the findings of this study may be obtained from the corresponding author upon reasonable request. Access will be provided for research purposes only, subject to institutional and ethical guidelines.

## Abbreviations

1080 Sprint: Motorized resistance device used to assess sprint mechanics
PS: Peak speed
PP: Peak power
PF: Peak force
SBJ: Standing broad jump
CMJ: Countermovement jump
CMJ JH: Jump height (CMJ-derived)
CMJ RF: Relative force (CMJ-derived) normalized to body mass
CMJ RP: Relative power (CMJ-derived) normalized to body mass
CoD: Change of direction
DJ: Drop jump
ES: Effect size
MDG: Microdosed training group
RSI mod: Modified reactive strength index from drop jump (jump height · ground contact time⁻¹)
TRG: Traditional training group
15-0-5: Change of direction test performed on the 1080 Sprint, consisting of Phase A (15 m acceleration followed by deceleration to a complete stop) and Phase B (5 m reacceleration)
