# Supplementary material for: The impact of microdosed plyometric training on speed and explosive abilities of football players during the pre-season
Source: BMC Sports Sci Med Rehabil. 2026 Feb 5;18:116. doi: 10.1186/s13102-026-01556-5 (PMC12973569; doi:10.1186/s13102-026-01556-5)
Supplement: Supplementary file 1 — Supplementary Material 1 [file 13102_2026_1556_MOESM1_ESM.docx]

| **Performance parameter** | **Within group Sensitivity ES (\|r\|)** | | **Between group Sensitivity ES (\|r\|)** |  |
| --- | --- | --- | --- | --- |
|  | **Traditional (n=12)** | **Microdosed (n=12)** |  |  |
|  |  |  |  |  |
| ***RSI mod*** | 0.92 | 0.64 | 0.06 |  |
| ***Jump height [cm]*** | 0.79 | 1.00 | 0.23 |  |
| ***Relative force [N·kg^-1^]*** | 0.15 | 0.03 | 0.06 |  |
| ***Relative power [W·kg^-1^]*** | 0.56 | 0.74 | 0.35 |  |
| ***Broad jump bilateral [cm]*** | 0.56 | 0.08 | 0.29 |  |
| ***Broad jump left [cm]*** | 0.74 | 0.21 | 0.42 |  |
| ***Broad jump right [cm]*** | 0.54 | 0.65 | 0.08 |  |
| ***Peak speed [m·s^-1^]*** | 0.91 | 0.90 | 0.07 |  |
| ***Peak force [N]*** | 0.64 | 0.82 | 0.01 |  |
| ***Peak power [W]*** | 0.76 | 0.90 | 0.00 |  |
| ***Time 15-0-5 [s]*** | 0.62 | 0.00 | 0.14 |  |
| ***Max acc phase A [m·s^-^²]*** | 0.18 | 0.23 | 0.17 |  |
| ***Max dec phase A [m·s^-^²]*** | 0.38 | 0.92 | 0.36 |  |
| ***Max acc phase B [m·s^-^²]*** | 0.77 | 0.74 | 0.08 |  |

**Supplementary Table S1**. Within-group and between-group sensitivity effect sizes (rank-biserial |r|).

**Note.** RSI mod = modified reactive strength index; acc = acceleration; dec = deceleration. Sensitivity effect sizes are reported as absolute rank-biserial correlations (|r|): Wilcoxon signed-rank tests for within-group pre–post change and Mann–Whitney U tests for between-group comparisons on change scores (Δ). Values are absolute magnitudes.
